# Oral Delivery of a Tetrameric Tripeptide Inhibitor of VEGFR1 Suppresses Pathological Choroid Neovascularization

**DOI:** 10.3390/ijms21020410

**Published:** 2020-01-09

**Authors:** Valeria Tarallo, Emanuela Iaccarino, Valeria Cicatiello, Riccardo Sanna, Menotti Ruvo, Sandro De Falco

**Affiliations:** 1 Istituto di Genetica e Biofisica ‘Adriano Buzzati-Traverso’—CNR, 80131 Napoli, Italy; 2 Istituto di Biostrutture e Bioimmagini—CNR, 80134 Napoli, Italy; 3 BIOVIIIx s.r.l., Department of R&D, 80142 Napoli, Italy; 4 ANBITION s.r.l., Department of R&D, 80128 Napoli, Italy

**Keywords:** AMD, CNV, VEGFR1, multimeric peptides, oral delivery

## Abstract

Age-related macular degeneration (AMD) is the primary cause of blindness in advanced countries. Repeated intravitreal delivery of anti-vascular endothelial growth factor (VEGF) agents has represented an important advancement for the therapy of wet AMD with significative results in terms of blindness prevention and partial vision restore. Nonetheless, some patients are not responsive or do not attain significant visual improvement, intravitreal injection may cause serious complications and important side effects have been reported for the prolonged block of VEGF-A. In order to evaluate new anti-angiogenic strategies, we focused our attention on VEGF receptor 1 (VEGFR1) developing a specific VEGFR-1 antagonist, a tetrameric tripeptide named inhibitor of VEGFR 1 (iVR1). We have evaluated its anti-angiogenic activity in the preclinical model of AMD, the laser-induced choroid neovascularization (CNV). iVR1 is able to potently inhibit CNV when delivered by intravitreal injection. Surprisingly, it is able to significantly reduce CNV also when delivered by gavage. Our data show that the specific block of VEGFR1 in vivo represents a valid alternative to the block of VEGF-A and that the inhibition of the pathological neovascularization at ocular level is also possible by systemic delivery of compounds not targeting VEGF-A.

## 1. Introduction

Aberrant ocular neovascularization is involved in many vision-threatening diseases including age-related macular degeneration (AMD), diabetic retinopathy (DR), central retinal vein occlusion (CRVO), retinopathy of prematurity (ROP) and corneal neovascularization. Among them, wet AMD accounts for about eight percent of all blindness worldwide and is the primary cause of blindness among the elderly in industrialized nations [1,2].

In the last years, anti-angiogenesis agents have revolutionized the treatment of ocular neovascular diseases [3,4]. Three anti-VEGF agents are currently available for therapy: ranibizumab and bevacizumab that specifically neutralize VEGF-A [5], and aflibercept, able to block VEGF-A, VEGF-B and placental growth factor (PlGF) [6]. Their delivery by repeated intravitreal injections blocks the growth of pathological vessels preventing blindness and, in many cases, restores vision.

Despite this significant clinical success, many patients do not attain significant visual improvement [7,8]. It has been shown that expression of VEGF in eyes is regulated by advanced glycation end products generated by protracted diabetes symptoms and that this can be causally associated with diabetic retinopathy, characterized by an increased retinal neovascularization due to the action of VEGF [9]. Adverse effects of VEGFA neutralization on multiple retinal cell types, widely reported in animal models [10,11], are observed in patients who have been treated with anti-VEGF drugs for several years [5,12,13]. Moreover, drug delivery by repeated intravitreal injections may generate devastating ocular complications. The most frequent are infectious endophthalmitis and intraocular inflammation. Ocular hemorrhage, intraocular pressure elevation and rhegmatogenous retinal detachment have also been observed [14], therefore, alternative or additional therapeutic anti-angiogenic strategies possibly coupled to different routes of administration are continuously sought.

VEGFR1 is the common receptor of the pro-angiogenic members of the VEGF family: VEGF-A, VEGF-B and PlGF [15]. It is also known as the high affinity receptor for VEGF-A since VEGF-A also recognizes VEGFR2, but with a KD increased by one order of magnitude [16]. The VEGF-A/VEGFR2 axis activates the main signaling pathway for the formation of new blood vessels from the pre-existing ones both in physiological and pathological conditions [17]. In parallel, several reports have highlighted the crucial function of VEGFR1 activation, mainly in pathological angiogenesis. Genetic ablation of VEGFR1 TK domain [18] or of VEGFR1 specific ligands, PlGF [19,20] and VEGF-B [21], gives rise to normal mice that show an inhibited pathological angiogenesis. Mirroring these studies, biochemical inhibition of VEGFR1 or PlGF, by the use of neutralizing monoclonal antibodies [22,23] or by small peptides [24,25] is effective in inhibiting the angiogenesis associated to several disease states, such as cancer, ocular neovascular diseases, inflammatory diseases, atherosclerosis and obesity [26].

To match the need of new therapeutic anti-angiogenic strategies, we developed a tetrameric tripeptide inhibitor of VEGF receptor 1 (VEGFR1), named iVR1 [27].

iVR1 binds to VEGFR-1, inhibiting the interaction with all three natural ligands with a half maximal inhibitory concentration (IC_50_) close to 8–10 µM. It is composed by unnatural amino acids that, together with the multimeric structure, confer high resistance to the degradation in biological fluids. iVR1 activity has been already fully characterized in vitro and in vivo. It specifically binds VEGFR1 and does not interfere with VEGFR2 activity, is able to prevent VEGFR1 phosphorylation and capillary-like tube formation of human primary endothelial cells, and also blocks neovascularization of chicken embryo chorioallantoic membrane induced by PlGF or VEGF-A [27]. In vivo, iVR1 suppresses tumor growth and neoangiogenesis in xenograft models of colorectal cancer to an extent similar to that exhibited by bevacizumab. It is able to synergize with the chemotherapeutic agent irinotecan, inducing a significant prolongation of survival similar to that observed with the combination of bevacizumab and irinotecan. Moreover, iVR1 delivered by intravitreal injection is also able to inhibit pathological angiogenesis in the preclinical model of wet AMD, the laser-induced choroid neovascularization (CNV) [28].

We have here evaluated the favorable option of administering iVR1 by a less traumatic route of administration for the therapeutic treatment of wet AMD. In the perspective of a systemic delivery of iVR1, we first performed a counter-ion exchange from trifluoroacetate (TFA), deriving from the chemical synthesis of the peptide (named here iVR1-TFA), to acetate, obtaining a new compound named iVR1-Ac. Replacement of TFA with acetic acid has also entailed a potency gain evaluated as a reduction of the IC_50_ of the inhibition of VEGF-A/VEGFR1 interaction in vitro of about five times. We have confirmed the inhibitory activity of iVR1-Ac in the preclinical model of wet AMD after intravitreal delivery and we have investigated whether the oral delivery by gavage could provide effective inhibition of laser-induced CNV.

## 2. Results

### 2.1. Synthesis of iVR1

**Figure 1:**
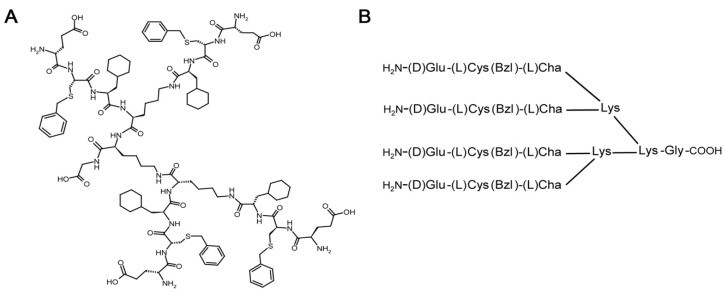

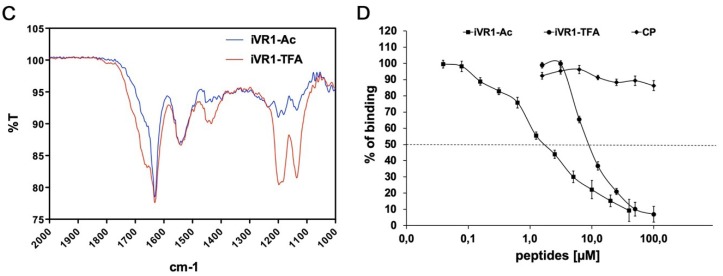
iVR1-Ac shows an increased inhibitory activity compared to iVR1-TFA. (**A**) Chemical structure of iVR1 tetrameric tripeptide that has a calculated molecular weight of 2362.02 g/mol. (**B**) Schematic representation of the iVR1. L-Cys(Bzl), L-cysteine(S-benzyl); L-Cha, L-cyclohexylalanine. (**C**) Overlay of FT-IR spectra in the spectral range between 1000 and 2000 cm^−1^ of the peptide before (red line) and after treatment with acetic acid (blue line). The spectrum collected on the acetic acid treated peptide shows that the bands characteristic of TFA at about 1145 and 1200 cm^−1^ are drastically reduced following repeated lyophilization in 0.1 M acetic acid. Some bands at around 1666 cm^−1^ were also strongly suppressed as consequence of the TFA removal. (**D**) Competitive ELISA for the binding of VEGF-A to immobilized VEGFR1. iVR1-Ac showed a decreased IC_50_ as compared to iVR1-TFA (1.94 and 9.35 µM, respectively) estimated as the concentration at 50% inhibition. Control peptide (CP) was inactive in the concentration range tested (up to 100 µM). Data are presented as the mean ± SEM of two independent experiments performed in triplicate.

iVR1 is a tetrameric tripeptide composed by unnatural amino acids having the following sequence and structure: [D-Glu-L-Cys(Bzl)-L-Cha]4-Lys2-Lys-Gly, whereby Cys(Bzl) indicates S-benzylated L-cysteine and Cha indicates cyclohexylalanine (Figure 1A,B). The peptide has been assembled by solid phase synthesis and removed from the resin using a mixture of TFA containing suitable scavengers, as previously reported [29]. The peptide has been obtained in a good yield (about 60% after purification) and in high purity (>95% as determined by HPLC analysis at 214 nm). Peptide identity has been assessed by LC–MS using an electrospray ionization time-of-flight (ESI-TOF) mass spectrometer coupled to HPLC. By virtue of the final TFA treatment and reverse phase purification the peptide is obtained as trifluoroacetate salt [29]. Since we have observed several times in previous studies [27,28] that TFA-peptide compounds are partially irritating the skin of the animal on the site of injection and given the known much higher toxicity of TFA salts compared to acetic acid, we have thus evaluated the option of using the peptide bearing the acetate as counter-ion instead of TFA, also considering that acetate is largely used in the formulation of therapeutic peptides.

### 2.2. FT-IR Characterization of Peptides Before and after Counter-Ion Exchange

Trifluoroacetate to acetate exchange is generally not a trivial operation. However, we tried to replace TFA by a triple step of peptide dissolution in acetic acid solutions and lyophilization. TFA removal was assessed by FT-IR spectroscopy comparing specific TFA IR bands before and after the treatment. We referred in particular to bands appearing as a shoulder at about 1670 cm^−1^ and two strong absorption bands at about 1135 and 1200 cm^−1^ [30]. These bands in proteins and peptides appear well above 1200 cm^−1^ [31], therefore they could be used as a good fingerprint to determine TFA amounts by comparing relative intensities. The data in Figure 1C shows that more than about 90% of the initial TFA content was removed by our treatment [30], suggesting that the method, with some improvements, could be used for replacing TFA with acetate in peptide samples.

### 2.3. iVR1Ac Shows Increased Inhibitory Activity Compared to iVR1-TFA

To verify whether the exchange of TFA with acetic acid could interfere with the inhibitory activity of iVR1, we performed a competitive ELISA using both compounds and as further control an unrelated peptide. iVR1-TFA, iVR1-Ac and the control peptide (CP) were used in competition for the binding of VEGF-A to VEGFR1. As shown in Figure 1D, iVR1-TFA was able to inhibit the binding of VEGF-A to VEGFR1 with an IC_50_ of 9.35 µM, whereas iVR1-Ac showed a greater competing activity with an IC_50_ close to 1.94 µM. The CP was unable to inhibit VEGF-A/VEGFR1 interaction. Thereby the counter-ion exchange determined an increase in terms of inhibitory activity of about five times, likely due to a partial improved solubility.

### 2.4. Intravitreal Delivery of iVR1-Ac Potently Inhibit Laser-Induced CNV

**Figure 2:**
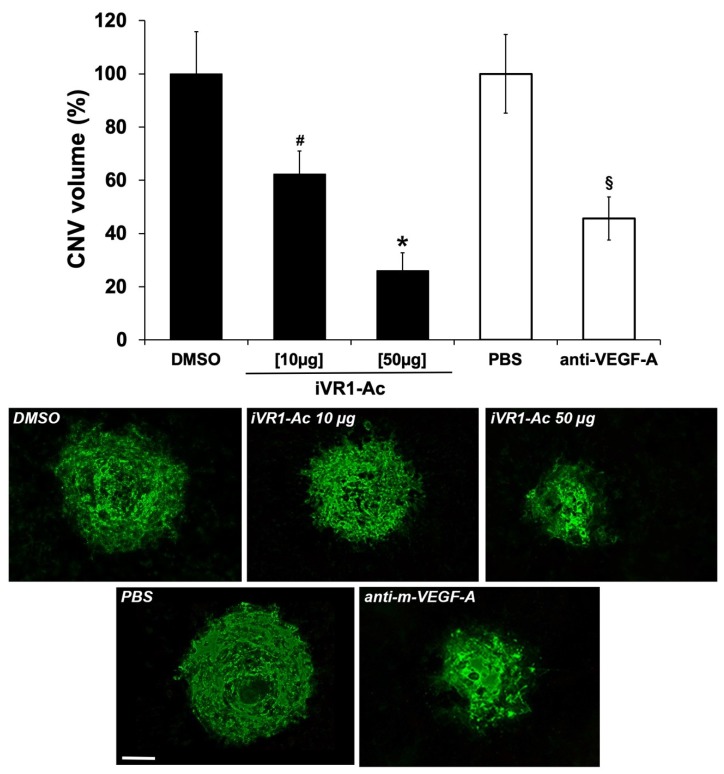
iVR1-Ac inhibits laser-induced choroid neovascularization (CNV) in a dose-dependent manner after intravitreal delivery. After 7 days from laser-induced damage, CNV volumes were measured by Isolectin B4 staining of RPE-choroid flat mounts. *N* = 5 mice per group. The following number of spots were analyzed: DMSO = 14, iVR1-Ac [10 µg] = 12, iVR1-Ac [50 µg] = 15; PBS = 10, anti-m-VEGF-A = 8. Data are presented as the mean ± SEM. * *p* = 0.0002 and # *p* = 0.0464 vs. DMSO; § *p* = 0.001 vs. PBS. On the bottom, representative pictures of CNV are shown. Scale bar: 100 µm.

Laser-induced CNV experiments were performed to verify the ability of iVR1-Ac to inhibit pathological neovascularization in vivo. Immediately after the induction of laser damage, single intravitreal injections of iVR1-Ac or of the vehicle (DMSO) and of anti-mouse VEGF-A polyclonal antibody used as positive controls, were performed. After seven days, CNV volume was evaluated by immunofluorescence analysis of retinal pigment epithelium (RPE) choroid flat mounts. Anti-mouse VEGF-A induced a strong and significant inhibition compared to PBS (−54.3%, *p* = 0.001 vs. PBS). iVR1-Ac was able to induce a dose-dependent inhibition of CNV. Indeed, at 10 µg it already induced a significant reduction of CNV (−37.8%, *p* = 0.0464 vs. DMSO) and become even stronger at the highest quantity delivered of 50 μg (−73.9%, *p* = 0.0002 vs. DMSO; Figure 2).

### 2.5. iVR1-Ac Delivered by Gavage Provides Effective CNV Inhibition

In order to look for alternative route of administration for treating wet AMD, we evaluated whether systemic delivery of iVR1-Ac by gavage was similarly effective. The administration of the peptide, and as control of the vehicle (200 µL each dose), started 12 h after the damage induced by laser and was performed over 7 days, two times per day, at 50 mg/Kg. This dosage was chosen based on previous data obtained in in vivo experiments for tumor studies [28]. 

**Figure 3:**
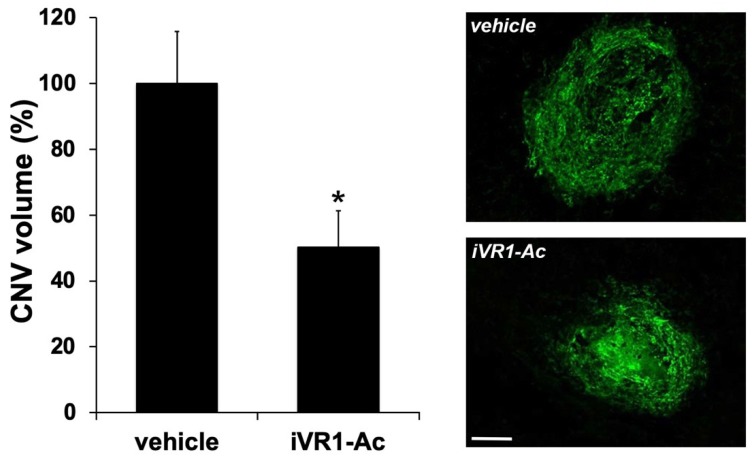
iVR1-Ac inhibited laser-induced CNV when delivered by gavage. After 7 days from laser-induced damage, CNV volumes were measured by Isolectin B4 staining of RPE-choroid flat mounts. *N* = 5 mice per group. The following number of spots were analyzed: vehicle = 10, iVR1-Ac = 20. Data are presented as the mean ± SEM. * *p* = 0.001 vs. vehicle. On the right, representative pictures of CNV are shown. Scale bar: 100 µm.

iVR1-Ac suppressed CNV by of about 50%, inhibiting pathological neovascularization (*p* = 0.001 vs. vehicle; Figure 3).

## 3. Discussion

Two main concerns affect the current anti-angiogenic therapies for ocular neovascular diseases: the side effects deriving from the prolonged block of VEGF-A and the tedious and the potentially dangerous practice of intravitreal injection. This last concern is also associated with the general reluctance of patients to be submitted to intravitreal punctures, most often accepted with worries and fright. 

Several data from preclinical models and patients show how detrimental can be the block of VEGF-A, and consequently of VEGF-A/VEGFR2 signaling, given its involvement also in physiological settings. VEGFR1 is also deeply involved in neoangiogenesis, however its activity is mostly restricted to pathological conditions. On this basis, we chose it as a privileged and more selective therapeutic target for angiogenesis inhibition. If the VEGF-A/VEGFR2 pathway is crucial for the stimulation, differentiation and migration of endothelial cells, as well as for the physiological homeostasis of vessels [32], the ability of VEGFR1 to drive neo-angiogenesis depends essentially on its wide pattern of expression and on its ability to drive survival, migratory, and cells recruitment signals [15,33]. Indeed, it is expressed on endothelial cells, where it has a role in vessel sprouting and growth [34], in mural cells, where drives their recruitment essential to stabilize nascent vessels [32], and in inflammatory cells that are recruited and activated at neo-angiogenic sites where they result crucial to further foster the vessel growth and stabilization [35].

The importance of VEGFR1 in pathological neoangiogenesis was also confirmed by our previous data. In the preclinical model of colorectal cancer [28], iVR1 was able to inhibit tumor growth and vascularization with an extent similar to that induced by bevacizumab, despite an IC_50_ close to 10 µM. It potently inhibited VEGFR1 phosphorylation in vivo and induced a strong decrease of monocyte-macrophages and mural cells recruitment. Here, we reported on a further advancement of knowledge on iVR1. We had indeed observed a considerable improvement of the peptide ability to inhibit VEGFR1 activity when the TFA counter-ion was removed or reduced from the formulation and replaced with acetate. Such improvement is reflected by the IC_50_ passing from about 10 µM to less than 2 µM. Intravitreal delivery of this new version of iVR1 in the preclinical model of wet AMD determines an inhibition of neovascularization to an extent never observed with anti-mouse VEGF-A antibodies. Together, these results demonstrated that in these in vivo preclinical models of diseases, the prevention of VEGFR1 activation was a valid and effective alternative to the block of VEGF-A. 

As anticipated, a major concern associated to the anti-angiogenic therapy for eye diseases is the delivery achieved by intravitreal injection of the drugs. Very few reports have so far described the delivery of anti-angiogenic drugs by oral administration for wet AMD. They are all referred to multitarget tyrosine kinase inhibitors (TKi; i.e., lenvatinib, pazopanib) mainly selected for their activity on VEGFR2 [36,37,38]. It must be underlined that the use of TKi substantially mirrors the block of VEGF-A achieved with current therapeutic agents but add further complications deriving from their multitargeting properties. It is therefore expected that this therapeutic strategy may have the dual side effects of prejudicing the physiological role of VEGF-A/VEGFR2 axis and suppressing the potentially beneficial activity of many other unrelated receptors targeted by the TKi, both at ocular and systemic levels.

Here we demonstrated that iVR1 was able to inhibit the CNV by 50% when delivered by gavage, displaying, for the first time at the best of knowledge, that inhibition of pathological neovascularization at ocular level was also feasible by systemic delivery of an anti-angiogenic peptide not inhibiting the VEGF-A/VEGFR2 axis. The dosage of 50 mg/kg was based on previous results obtained in tumor models, in which iVR1 was delivered by intraperitoneal injection every other day at 50 mg/kg for 14 days or at 25/mg/kg for 80 days [28]. This dosage regimen had not evidenced any gross side effects or sign of toxicity. These previous data and those we reported here allowed us to speculate that the systemic block of VEGFR1 could determine low or negligible side effects deriving from its minor role in physiological processes.

The prevalence of wet AMD is destined to increase in the next years as a consequence of exponential population ageing [1]. Accordingly, the request of new anti-angiogenic drugs, even better if delivered by routes alternative to the intravitreal injection, will consequently increase expanding enormously the interest for this class of molecules. Clearly, to propose iVR1 for further therapeutic studies, iVR1 toxicity, pharmacokinetic and pharmacodynamic studies at ocular and systemic levels are needed. In this perspective, analogues of iVR1 obtained from the iVR1 chemical structure have already been identified and will be soon characterized for their anti-angiogenic properties to assess the therapeutic potential. 

## 4. Materials and Methods

### 4.1. Peptide Synthesis

All chemicals for peptide synthesis were from commercial sources and used without further purification unless otherwise stated. Solvents, including acetonitrile (CH_3_CN), dimethylformamide (DMF) were purchased from Sigma Aldrich (Milano, Italy). Acetic acid, *N*,*N*-Diisopropylethylamine (DIPEA), piperidine, and TFA were also from Sigma-Aldrich. Protected amino acids and the coupling agents OxymePure^®^ (ethyl 2-cyano-2-(hydroxyimino)acetate, called Oxyma Pure) and *N*-*N*′ diisopropylcarbodiimide (DIC) used for peptide synthesis were from IRIS Biotech (Marktredwitz, Germany). The peptide was prepared as previously reported [27,28], with some modifications. In particular as coupling and deprotection agents we used Oxyma/DIC/DIPEA and 40% piperidine in DMF to improve the final yield and to obtain purer products. The crude material was characterized by LC–MS [29] and purified by preparative RP-HPLC using a gradient from 20% solvent B (CH_3_CN, 0.1% TFA) to 80% solvent B over solvent A (H_2_O, 0.1% TFA) in 20 min, monitoring the eluate at 214 nm. The purified material was collected, lyophilized, and characterized for purity and identity by LC–MS using the conditions previously reported [29].

### 4.2. FT-IR Characterization of Peptides Before and After Counter-Ion Exchange

To assess the removal of TFA, purified peptides were characterized by FT-IR spectroscopy. To remove the TFA the peptide samples were dissolved in 0.1 M acetic acid in water and lyophilized for at least 24 h. The treatment was repeated at least three times. To assess removal of TFA, solid samples were analyzed before and after treatment by FT-IR spectroscopy using a Jasco FT/IR 4100 spectrometer (Jasco Europe, Cremella, Italy). Samples were ground into a fine powder and analyzed by FT-IR following the attenuated total reflection (ATR) technique. In these experiments a dry peptide film, from which most of the free solvent water was removed, was prepared on the ATR crystal. Then the characteristic peaks of IR transmission spectra were recorded at a resolution of 4 cm^−1^ over the wavenumber region of 400–4000 cm^−1^. 

### 4.3. Competitive ELISA for VEGF-A/VEGFR-1 Interaction

The competitive ELISA based assay was performed by coating on 96-well plates a recombinant form of VEGFR-1 (R&D Systems, Minneapolis, MN, USA) at 0.5 µg/mL in PBS, 100 µL/well (the same volume was used for all subsequent steps), 16 h at room temperature. The plate was then blocked for 3 h at RT with 1% bovine serum albumin (BSA). Recombinant form of VEGF-A (R&D Systems) at 5 ng/mL in PBS containing 0.1% BSA, 5 mM EDTA, 0.004% Tween 20 (PBET) and 4% DMSO, was added alone or mixed with peptides at a concentration ranging between 0.04 and 100 µM, and incubated for 1 h at 37 °C followed by 1 h at RT. A biotinylated anti VEGF-A polyclonal antibody (R&D Systems) diluted in PBET at 150 ng/mL, was added to the wells and incubated for 1 h at 37 °C followed by 1 h at RT. A solution containing an avidin and biotinylated horseradish peroxidase (HRP) macromolecular complex was prepared as suggested by the manufacturer (Vector Laboratories, Burlingame, CA, USA) and added to the wells and incubated for 1 h at RT followed by the HRP substrate composed of 1 mg/mL of ortho-phenylenediamine in 50 mM citrate phosphate buffer pH 5, 0.006% of H_2_O_2_, incubated for 40 min in the dark at RT. The reaction was blocked by adding 30 µL/well of 4 N H_2_SO_4_ and the absorbance measured at 490 nm on a microplate reader (BenchMark, Biorad, Hercules, CA, USA).

### 4.4. Animals

C57Bl6/J were purchased from Charles River. Animal experiments were run in accordance with European directives no. 2010/63/UE and Italian directives D.L. 26/2014, and were approved by the Italian Ministry of Health (authorization no. 695/2015-PR of 17 July 2015). For laser-induced CNV, anesthesia was performed by intraperitoneal injection of 100 mg/kg ketamine hydrochloride and 10 mg/kg xylazine. Pupils were dilated with topical tropicamide (1%, Visupharma, Rome, Italy).

### 4.5. Choroidal Neo-Vascularization Model: Intravitreal Delivery

Laser photocoagulation was performed on 6–8 weeks old C57Bl6/J mice (*n* = 5 per group), using a 532-nm laser (Meridian, Thun, Switzerland) connected to the Micron IV apparatus (Phoenix Research Labs), Pleasanton, CA, USA). Eyes in which a massive sub-retinal hemorrhage developed after laser induction of CNV were excluded from the analysis. Immediately after the laser application, 10 or 50 µg of iVR1 in 1 µL of vehicle (DMSO) were intravitreally injected with a microsyringe (Hamilton Italy, Agrate Brianza, Italy) carrying a 33-gauge needle. As positive control of inhibition, 2 ng of anti-mouse VEGF-A polyclonal antibody (R&D Systems) in 1 μL of PBS were intravitreally delivered. Of vehicles (DMSO or PBS) 1 µL was delivered to the contralateral eyes.

### 4.6. Choroidal Neo-Vascularization Model: Oral Delivery by Gavage

Laser photocoagulation was performed on 6–8 weeks old C57Bl6/J mice (*n* = 5 per group). iVR1 was dissolved in DMSO and daily mixed with food thickener Nutilis (Nutricia, Hoofddorp, The Netherlands) to have a final concentration of 6.25 mg/mL of iVR1 in 4.25% Nutilis, 10% DMSO. Of peptide preparation (50 mg/Kg) 200 µL was administered by gavage using a syringe carrying a rounded tip 20-gauge needle, starting 12 h after laser damage, over 7 days, two times per day. To control animals, the same volume of vehicle was delivered.

### 4.7. Choroidal Neo-Vascularization Volume Quantification

Seven days after laser injury, eyes were enucleated and processed to isolate eye-cups that were stained with 0.7% FITC-conjugated *Griffonia simplicifolia* Isolectin B4 (Vector Laboratories). Afterwards, retinae were removed and RPE-choroid were flat mounted by four incisions under dissecting microscope and then mounted with Vectashield. CNV were visualized under Leica DM6000 fluorescent microscope and horizontal optical sections were obtained at every 1-μm step from the surface to the deepest focal plane. The CNV volume was measured summing the whole fluorescent area of each optical section with ImageJ software. 

### 4.8. Statistical Analysis

Results are expressed as mean ± SEM, with *p* values < 0.05 considered statistically significant. Differences among groups were compared by the Student’s *t* test (two-tailed) or one-way ANOVA.

## 5. Patents

Patents resulting from the work reported in this manuscript: Italian patent n. 102018000008507 filed on 11 November 2018, entitled: “Peptidi ed usi medici correlati”.

## Abbreviations

AMD	age-related macular degeneration
CNV	choroid neovascularization
CRVO	central retinal vein occlusion
DR	diabetic retinopathy
iVR1	inhibitor of VEGF receptor 1
ROP	retinopathy of prematurity
RPE	retinal pigment epithelium
TFA	trifluoroacetate
TKi	tyrosine kinase inhibitors
VEGF	vascular endothelial growth factor
VEGFR1	vascular endothelial growth factor receptor 1
VEGFR2	vascular endothelial growth factor receptor 2
